# A Methodological Model for the Promotion of Sexual Corporeal Health and Self-Care

**DOI:** 10.3390/ijerph18095034

**Published:** 2021-05-10

**Authors:** Jimena Silva Segovia, Pablo Zuleta Pastor, Estefany Castillo Ravanal

**Affiliations:** 1 Dirección de Investigación, Postgrado y Transferencia Tecnológica, Universidad de Tarapacá, Arica 1000000, Chile; 2 Facultad de Ciencias Sociales, Escuela de Psicología, Universidad Bernardo O’Higgins, Santiago 8320000, Chile; pablozuletapastor@gmail.com; 3 Facultad de Humanidades, Escuela de Psicología, Universidad Católica del Norte, Antofagasta 1240000, Chile; castilloravanale@gmail.com

**Keywords:** sexual self-care, body, methodology, maps, gender

## Abstract

The objective of this article is to contribute to sex education with a methodology that facilitates subjective expression through the body in its different experiences. For that, we propose an intertextual model of sexual self-care that focuses on gender and rights. This work strategy stimulates the emergence of meanings and discourses embodied in a protagonist’s body. These procedures are applied in interactive workshops, where the experience narrated, written and graphed on one’s own body and sexuality is articulated. Based on this amalgam, a body map is drawn that illustrates a geography of interpersonal relationships. In the process, the importance of gender mandates, coming from meaningful figures, is understood. From these findings it is possible to self-analyze experiences that emerge from the intrapsychic levels weaved with sociocultural and emotional experiences, which opens opportunities for the deconstruction of hegemonic positions. The relationship that develops between the person who produces the intertextual map of the body and the person who orients the process is dialogical in that the notions of authorship, agency and subjective autonomy are recognized, which increases the possibilities of redefining gender’s position in social relationships and provides a strategy for educational programs considered from the protagonist’s perspective. It is concluded that this model facilitates the process of corporeal self-care in that it strengthens autonomy through the recognition of authorship and agency, strengthening the redefinition of a gendered position in social relationships, providing a strategy for educational prevention programs and the promotion of sexuality.

## 1. Introduction

Sexuality, according to post-structuralizing critical focuses, has been redefined as a social construction related to multiple and intricate ways of expressing and experiencing desire, emotions and emotional relationships. Its dynamic includes biological, physiological and morphological aspects of the body that provide the prior conditions for the social construction of sexuality [1,2]. Based on this focus, sexuality emerges and is transformed into diverse cultural contexts, where a series of characteristics interact, such as psychological, age, socioeconomic, gender, ethnic, and sexual orientation factors, among others, that require attention when developing sex education strategies.

With sex education based on rights, it is necessary to consider the concept of sexual health, which is related to the integration of the corporal, emotional, intellectual, social and cultural aspects of men and women and their particularities, to satisfactorily enrich the working model and to strengthen the expression of personality without restrictions [3]. Sex education seeks to strengthen a capacity for enjoying and expressing a healthy sexuality exempt from coercion, violence or gender discrimination, which constitutes the basis for mental health. Sex education also promotes sexual health based on the planning of pregnancies and protection from sexually transmitted diseases to develop corporal self-care.

From its foundations, the sex education model that is proposed contributes to a recognition of rights and the development of satisfactory personal relationships, which means elevating the capacities for expressing and feeling pleasure and strengthening an independent self-concept that promotes autonomy in decision-making regarding sexual relations. This model can strengthen decision-making in sexual life, avoiding damage, and choosing the time, space and moment without pressure and risks to health or psycho-sexual stability.

In this same theoretical perspective, work is conducted under the concept of reproductive health, which links to rights articulated with a state of physical, mental and social wellbeing, not just based on the absence of illness or discomfort. This work also includes the promotion of the right to have access to information, fertility planning methods and integral health services for women and men.

It has been considered in these foundations that biographically, men and women from 15 to 19 years of age are at a stage in life in which they have diverse characteristics. Some feel invulnerable, turn themselves over to emerging emotions and are moved by their immediate desires; others are moved by love, and some also give in to their partner’s pressure. In these experiences, without sex education with a gender and rights perspective, they find themselves with limited tools for organizing their sexual initiation or deciding on its timing. This is observed in the statistical data and in the qualitative information, where pregnancy and sexually transmitted diseases (STDs), including HIV/AIDS (Human Immunodeficiency Virus/Acquired ImmunoDeficiency Syndrome), constitute threats to their lives and plans.

The urgent need to develop strategic and close sex education models is linked to worldwide data that show that as of June 2014, 36.9 million people live with HIV [4] (As 2030 approaches, the world prepares to face the challenges posed by the ambitious Sustainable Development Objectives. The World Health Organisation is preparing three worldwide strategies for the health sector to face HIV/AIDS, viral hepatitis and sexually transmitted diseases (http://www.who.int/mediacentre/factsheets/fs323/en/ accessed on 10 March 2021)). According to “Ending Aids 2017”, Chile is the Latin American country with the largest number of new cases between 2010 and 2016. In total, 61,000 people are living with the virus in the country, of which 69% are informed of their situation and 53% are receiving some type of treatment. With a 34% increase, Chile is positioned above Guatemala, which has 23% of the new cases, followed by Costa Rica with 16%. Of these new cases, 4717 are women [5]. A concern related to these data is that the latest survey taken by the National Youth Institute (INJUV by its Spanish acronym) [6] revealed that 29.2% of young people did not use any method of prevention during their last sexual relations, because they indicate that they do not like “any of the methods I know”. In addition, 27.6% did not use a condom and argued that they are part of a stable couple. In young people aged 15 to 19 years, only 50% used a condom during their first sexual relations [6].

Fourteen countries have achieved HIV 90-90-90 treatment goals (90% of people who live with HIV are aware of their serological HIV status; 90% of those who are aware of that status have access to treatment; and 90% of the people in treatment for HIV have a suppressed viral load). Among them is Eswatini, which has one of the highest rates of HIV prevalence in the world, with 27% in 2019, and which, having surpassed its goals for 2020 is on the way to the 95-95-95 goals set for 2030 (Joint United Nations Program on HIV/AIDS, UNAIDS, 2020) [4] (https://www.unaids.org/es/resources/presscentre/pressreleaseandstatementarchive/2020/july/20200706_global-aids-report accessed on 10 March 2021).

## 2. Gender in Education

To contribute to a healthy sexual life in the population, the educational contents need to include body and gender, body and eroticism, body, affectivity and love. Therefore, it is also necessary to analyze the role that the dominant protagonist has in the school discourse in gender construction in childhood and youth. In these analyses, the reproductive nature of inequalities is identified in the classroom, texts and programs, and recognizes the incorporation of a gender order in the guidelines, values and norms of daily practices that places the construction of masculinity and femininity in radical opposition [7].

Nevertheless, despite constituting a space for a reproduction of inequalities, it has been demonstrated that receiving education means acquiring cultural capital such as knowledge that leads to the transformation of inequality through constant dialogue and the cultivation of an awareness of rights [8]. According to the analysis by Stromquist [9], education is an opportunity for contact with different ideas, knowledge with broad contexts, revolutionary possibilities, strategies and visions to analyze the subject’s experiences.

Sharing methodologies and critical and participative views contributes to subjective independence. For this subjective independence, it is necessary for the contents and strategies to pass through a process of deconstruction of the old ideas on teaching and to incorporate the analysis of gender distinctions in their supporting contents and context to facilitate the progress of new thoughts [10,11]. Different studies have compared the results of the sexual health of young people in the Netherlands with the sexual health of young people in other developed countries. In this review, it has been concluded that integral education in sexuality is one of the key factors that contributes to positive results in sexual health [12].

In other educational research, the findings have identified that in the school dynamic, the keys to the construction of hegemonic masculinity are conserved [13,14,15]. The findings reviewed indicate that in industrialized countries that have progressed in gender equality, polarized models are still found in primary and secondary education schools, in which boys dominate the classroom and the educator’s attention, while the girls’ contributions can become undervalued. It has been observed not only that sexual harassment is identified as an unresolved problem in young people’s relationships but also that differentiations persist between men and women in the election of areas of study [9,16,17].

We also reviewed the study by Allen [18] in which she talks with young people from New Zealand aged 16 to 19 years who are positively and legitimately positioned as sexual subjects and who express their opinion on the programs that emphasize the reduction in the negative results of sexual activity. With the findings, it is proposed that giving more weight to young people’s points of view would mean progressing toward effective sex education. This implies an advance in rights for their sexual health and wellbeing.

In Chile, the overall educational system is characterized by being structured as an institution that reproduces social gender differences according to socioeconomic level and educational quality that extend from prior family and cultural contexts to the educational trajectory, based on a society with overall inequalities [19]. In a model of socioeconomic and gender hierarchies, the students who belong to the educational system but who have reduced cultural capital as part of their social disadvantages correspond to the category “included in the system but not educable”; therefore, education in sexual and reproductive rights is not in their curriculum [19].

Accordingly, the students who have a better endowment of human capital and cultural capital resources have a greater likelihood of obtaining good educational results and access to more informed sex education to eclipse the social gender effects and obstacles to realizing their future plans, such as unplanned pregnancies or high-risk illnesses [6,20]. We refer to social (habits, knowledge and formed attitudes), not natural, endowments that are determined by the socioeconomic or cultural level of the family to which each student belongs based on which segregations in the educational system are produced [21].

## 3. Recover the Discourse of the Body for Sex Education

Since the 1980s, with the linguistic turn, a central role was granted to language in the construction of the world, and it also relegated the body to its material dimension. This was the object of strong criticism by post-Marxist feminist thought and the Foucaultian analysis regarding power mechanisms. The authors of these theoretical perspectives have therefore invited us to situate the discussion in a space that is not outside the body itself.

Nightingale and Cromby [22] defend that the body is an intimate place where nature and culture meet, so the centrality deposited in language leaves out aspects that are precisely related to the materiality of the social world. Possibilities for action are denied and the regimes of power that constrain the human experience are forgotten.

For this reason, Foucault in his analysis emphasizes materiality in the genealogy of power and the mechanisms of the subjectivation of the human experience. This process has been made more complex by feminist thinkers such as Rubin [23] who proposed that the socialization of gender would also result in a split between the sexes and generate a gender binary in the symbolic sphere that will govern behaviors. Preciado [24], in turn, places the responsibility for implementing the discipline of the sexually gendered body on gendered technologies.

The impact of this research is that a need is envisaged to recover the corporal experience not only as a perspective of analysis to understand the social world but also as a subjectivation of gender [25] that enables knowing identities and positioning the body not just as an object territory but rather as an agent that produces and reproduces the meanings that have been incorporated in the gender socialization process.

## 4. The Model Is Developed below, with Its Procedural Application Proposal and Levels of Analysis

The principal objective of the intertextual Maps model is to contribute to the recognition of cultural, subjective gender experiences and how they are inscribed in the body, leaving discursive marks and restrictive mandates; as well as contributing to empowerment as a process of autonomy and corporeal self-care.

The scientific literature shows inconsistencies between the way in which the corporal scheme and the corporal image are defined. The oldest term is that of corporal scheme, which originated in the early 20th century. The first person to talk about corporal scheme was Bonnier (1905) who defined it as a topographical figuration of corporality [26].

The concept of Corporeal Maps started to be disseminated as a technique with some applications, such as the Bach Flower System, developed by Dietmar Krämer and Helmut Wild, in the year 2000 in Argentina. This system enables everyone to be experts and to approach the body from a health perspective alternative to that of Western, academic medicine, seeking wellbeing. With a different focus, the Mask Institute of the University of Buenos Aires published the Phantasmatic Corporal Map by Mario Buchbinder and Elina Matoso (2011), with a prologue by David Le Breton. These authors propose an intervention device created, among other purposes, for the institutional diagnosis denominated Institutional Phantasmatic Map (IPM) (Stopiello, 2011) [27,28]. These forms of application offer this technique within the diagnostic logic [29].

Scientists from the University of Aalto confirmed that every emotion awakens reactions in certain zones of the body, and that this happens with people from very different cultures. Therefore, the authors of the work, which was published in the PNAS journal of the National Academy of Sciences of the United States, conclude that this physical, emotional map has biological bases and is universal (https://www.bbc.com/mundo/noticias/2014/01/140102_ciencia_mapa_corporal_emociones_np accessed on 10 March 2021).

**Figure 1:**
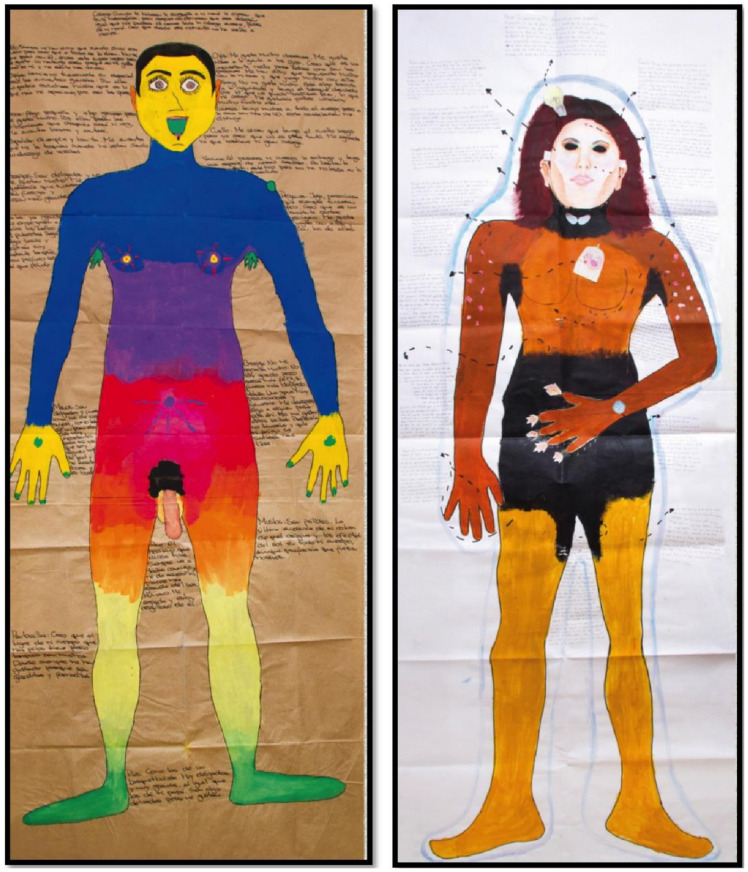
Examples of intertextual maps of the participants.

Unlike the foregoing, the proposed model of intertextual maps of the body does not seek to promote a diagnostic process or medical intervention, but rather to stimulate the emergence of meanings and discourses of the subject’s biography embodied in a protagonist body, with the objective of facilitating the expression of suffering, discomfort, or acknowledgment. Its procedures seek to articulate knowledge in a co-construction of written and oral accounts and autobiographical graphs with which a geography of the corporeal experience is elaborated based on interpersonal relationships with meaningful figures and a self-analysis of experiences that emerge from intrapsychic levels, weaved with the sociocultural and affective scenarios where the selected events occurred. The relationship that results between the subject who produces the corporeal map, and the researcher is dialogical, such that the concept of authorship is recognized in the person who elaborates the corporal map, highlighting the subject’s agency and autonomy in the production of knowledge and truth. The process described above can be summarized in Figure 1.

With the intertextual maps of the body, concerns are addressed regarding its sense and meaning, and its corporality expressed with language, which is interweaved in the biography of each subject. Considering the biographical experiences embodied, the concern for the sense of the body is one of the most enigmatic and persistent questions that crosses each existence, although the flesh deals with resistance and unknowns regarding where social power circulates [30]. Therefore, a corporeal map is proposed to elaborate a material anchor that represents what is corporeal, namely, flesh, bones, blood, and all of their systems amalgamated with symbolic references.

The model would enable reclaiming the subject’s agency: this text is mine, because this body is mine, subverting fastening mechanisms of the semiotic–material experience that are characteristic of scientific practice, such as the silencing of the subject who is speaking, the coding of subjectivity in categories and the subordination in the process of interpreting the experience of others. Therefore, through this model a way is opened for the body “to insist on being”, which means that “the body is and has its way despite the power and gender stereotypes” [30].

Based on these reappropriating acts of the corporeal being, the subject and the researcher accede to an understanding of the mandates, gestures, attitudes and symbols that articulate the social power, that subordinate the subjects and that are manifested in their sociosexual practices. These practices are organized as standards that the subjects, based on their autonomy, can oppose or interpret according to their biography and the decisions that they make [31].

This position seeks to contribute to sex education and proposes a methodological model oriented toward the study of the body based on its symbolic value. This model is set in the framework of the biographical models and critical theories and seeks to recover people’s psychosocial and contextual dimensions. In this sense, and following Pujadas [32], epistemological ruptures are provoked that lead social scientists to sources of social knowledge that examine in-depth what people and groups represent with their bodies.

## 5. Intertextual Maps of the Body for Sex Education Based on Gender and Rights

The concept of Corporeal Maps started to be disseminated as a technique with some applications, for example, in the Instituto de la Máscara of the Universidad de Buenos Aires, which published the Phantasmatic Corporal Map of Buchbinder and Matoso [33], introduced by David Le Breton. These authors propose that an intervention device be created for, among other purposes, the institutional diagnosis called the Phantasmatic Institutional Map (PIM) [28,34,35,36]. These forms of application offer this technique as part of the diagnostic and intervention logic that enables preparing a set of knowns under an arrangement of asymmetry of powers regarding the physical and mental health of the body based on expert knowledge (see Figure 1).

Unlike the above, with the proposed model of corporeal maps under the biographical method, a diagnostic or intervention process is not desired. In contrast, the emergence of meanings and discourses embodied in a protagonist body of the subject’s biography is stimulated. Its procedures seek to articulate knowledge in a co-construction of writing, verbal relation and autobiographical graphs with which a geography of the corporal experience is prepared based on interpersonal relationships with significant figures and a self-analysis of experiences that emerge from intrapsychic levels weaved with sociocultural and affective scenarios where the selected events occur. The relationship that is produced between the subject who produces the corporal map, and the researcher is dialogical such that the notion of authorship is recognized in the person who prepared the corporal map, which emphasizes the subject’s agency and autonomy in the production of the knowledge and truth. The process described above can be summarized in the following schematic.

### 5.1. Organization of the Group

The selection of the participants for the application of this model does not have restrictions of gender, age, or socioeconomic level. The main consideration is objective and thematic, which will be addressed in the workshops for forming the groups (either by the characteristics of the group or the topic to be addressed), for example, by groups (mixed or separatist) of university students, women heads of households, sex workers, male miners, etc., or by topic: embodied sexual violence, sexuality and comprehensive sexuality education, corporal self-care, (groups that will guide the main topic more than the characteristics of the group). Based on this, there will be cohesion among the participants. What is enriching about the methodological model is the heterogeneity of the group, which is considered a value of the investigative intervention process.

To execute the maps, the participants are required to: (a) express their willingness and availability to work in the process of corporal reappropriation and subjective autonomy based on the recovery of autobiographical events; (b) achieve continuity and regularity to participate in all the sessions; and (c) be willing to sign an informed consent form for the use of the autobiography, corporal map and narratives in the biographical research process.

This methodological model began to be developed in 2005 and to date, the processes and procedures have been conducted with approximately 300 people from 20 to 50 years old in workshops and courses [21].

### 5.2. Preparation of the Lifeline

As a tool, the lifeline is a primary step for organizing events in memory and is also a resource for easily finding these events if we need them.

In organizing a lifeline, it is sought to open the subjective memory process, connecting their life as a microstructure to the dynamic of continuous changes in the macrostructures, which contribute to documenting the distinctions in the trajectories between the life of a man and that of the relating woman [37,38].

The lifeline is organized as a graph in which a person locates the knots of events that for this person, are the most relevant in a period of life; it situates them so that they show sets of biographical processes in time sequences. Each represented knot enables identifying the tracks of biographical events, which provide an account of the processes situated in sociopolitical contexts (economic, political, and sociocultural).

### 5.3. Autobiographical Writings

For Lejeune and Boaert, (2003) [39] the autobiography is a retrospective account, in prose, that an individual makes of one’s existence, putting the accent on one’s own life, and focusing on the history of one’s personality. In this regard, as George May has suggested, placing an emphasis on one’s own existence does not exclude the fusion of the historical and individual planes [40].

**Figure 2:**
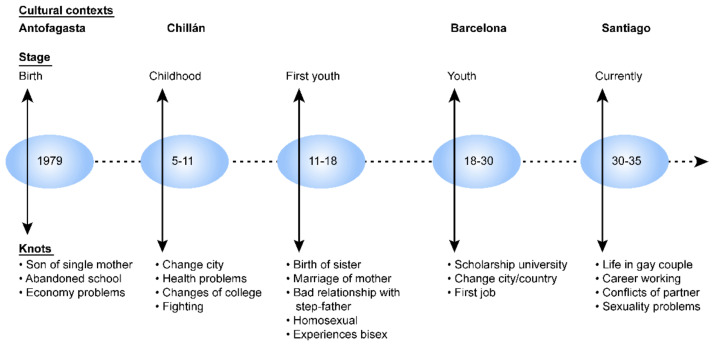
Example of the preparation of a lifeline, as instructed in courses and workshops.

Writing is initiated based on the production of lifelines (see Figure 2). Through reflexive questions, the participants are stimulated to open the biographic knots, and events are articulated in the context in which they occurred; meanwhile, the persons involved are identified, and the affections are displayed.

Possibilities arise in this autobiographical narrative for self-interpretations of interpersonal relationships with significant figures (for example, sexual, affective, caring, and punishment, among other relationships) and an analysis of the sociocultural and affective scenarios where the significant experiences that have been anchored in the processes associated with gender mandates have occurred.

This process of reflexivity stimulates the emergence of emotions from a microsocial focus of the social context and of the affective and gender relationships in the construction of the protagonists, thereby facilitating levels of understanding regarding conflicts, elections, ruptures, and tensions between the subjects and the significant persons from their environment, among other people.

As a recommendation, the narratives that emerge can be taped or written so that the expression of groups with reading or writing difficulties can be benefitted. This approach also benefits the reflexivity of this dialogical methodology that returns to the narrative to find new interpretive lines of the experience based on the process that each subject expresses.

### 5.4. Relating What Is Written

In systematized experiences with over 200 intertextual maps, with men and women from 15 to 60 years of age, situated in diverse cultural contexts, we have observed that in the process of remembering their positive and negative biographical events, gradually and accompanied by the group, this constitutes support. The person discovers a level of reflection regarding his or her capacity to initiate a transformation in the here and now. Subsequently, in that reordering, making sense of their story and portraying it in a map-body that is graphed and signified as her own, can recognize pains and options, with the echo of others like her. Therefore, the composition of the group and the continuity of the work until the end is fundamental. This way, stimulating that willingness to know about oneself strengthens a desire with which the subject can continue empowering herself or give up. However, there was already a transformation.

Each narration offers horizontal, vertical or circular entries to lived experiences that are represented over and over in the memory to form a part of human practices [41,42]. This part of the procedure is more spontaneous since the fragments to be narrated are freely chosen. This phase involves an effort to make sense of the past, the present, and the contents associated with the biographical project or what its reformulation or relaunching means.

It is important that the participants develop a posture during this phase regarding their experiences of pain or suffering and the experiences that were satisfactory. This posture promotes their empowerment, whether based on the self-recognition of their potentialities or the self-interpretation of the transformation options that the subject possesses.

The role of the researchers who accompany this phase, as monitors or guides, is to actively listen, facilitate the manifestation of vital experiences, and participate with the group in the interpretations.

### 5.5. Corporal Intertextuality

In this stage, the accounts, conversations, lifelines and autobiographies are gathered and are available to feed the corporal map. Therefore, this stage is the moment in which the biographical knots have been opened for recovery, reflexivity and putting into text. This phase is characterized by its level of symbolic density, since from here, a representation of the subjects is constructed that offers an intertextual interpretation of the construction of a corporal biography.

At this point in the process, work is performed under the criterion of willingness according to the application that the researcher, monitor or guide decides to give to the model.

This phase can be started with an instruction such as “We will draw symbols, words or messages that represent not only your body but also the experiences that you decide to work on”.

Each instruction should be adapted to the work group. Then, at this time, the paper or cloth of the actual size of the person’s body is prepared, and they are left free to use their creativity regarding the colors and materials for the applications or textures. Then, the participants are organized in pairs, and the contour of the body is drawn in the position most comfortable for the participant.

Finally, the symbolic elements of the experience with the body are represented, such as the characteristics of the self-image. On another level of representation, the different discourses that originate from the social image constructed based on diverse beliefs are recorded. These discourses seek to facilitate the expression of embodied cultural inscriptions. Finally, with the set of symbolic elements, work is conducted on the subjective recovery process.

It is pertinent to make explicit that although the model raises biographical questions and expressions of emotions, it does not perform clinical work from a diagnostic standpoint.

### 5.6. Close-Out Phase

**Figure 3:**
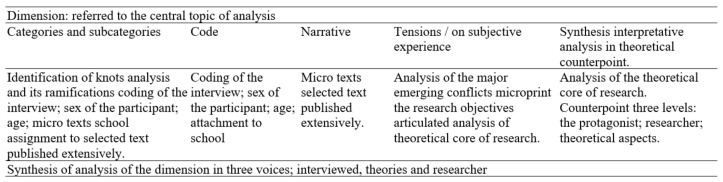
Example matrix narrative analysis. Source: Author.

**Figure 4:**
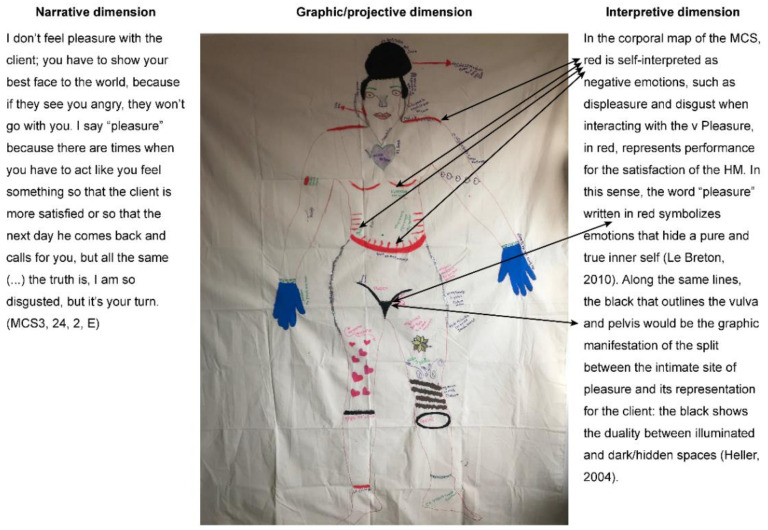
Example of a participative intertextual analysis and its three dimensions. Source: Self-created. Own elaboration. Dimension narrative = Narrative dimension. I don’t feel pleasure with the client; you have to show your best face to the world, because if they see you angry, they won’t go with you. I say “pleasure” because there are times when you have to act like you feel something so that the client is more satisfied or so that the next day he comes back and calls for you, but all the same (…) the truth is, I am so disgusted, but it’s your turn. (Women sex worker, 24, 2, E). Dimension Grafico/proyectiva = Graphic/projective dimension. Dimension interpretative = Interpretive dimension.

The detailed work of preparing the corporal map is performed alone, in a dialogue with oneself and one’s own self-interpretations. For this reason, in the close-out stage, a space is generated where the group and the subject voluntarily decide to share their experience. This is a process of intersubjective reflexivity, where the participants have generated opportunities to be acknowledged and to work with some obstacles that hinder their wellbeing and quality of life. The subjects engage in dialogue on their findings under a group methodology. The methodology enables discussing and interpolating their own findings as graphed on the map (see Figure 3 and Figure 4).

The narrative dimension comprises the recovery of spaces of memory and their interpretations, and these spaces are organized in knots or significant biographical conglomerates. This dimension is subdivided into (a) normative discourses on the body and restrictions, punishments, and mandates of beauty, aesthetics and gender [43,44] and (b) practices linked to what occurs at the topographical level of the body, such as the use of biotechnologies, medications, and corrective or aesthetic medical devices, among others.

With the analysis of these two interpretive levels—discourses and practices—it is possible to visualize discrepancies between theoretical discourses and subjective practices, especially regarding the dominant ideas on the processes of the gender socialization of the subject.

The corporal maps thereby enable producing knowledge that reveals that the subject is an active and ongoing transformer of social norms, which feeds the arguments against excessive linearity of classical conceptions of the socializing processes, which sustain that there is “a more or less direct link between the introjected norm and behavior” [43].

The graphic dimension consists of representations of self-image and social image interpreted by each subject as their corporal “geography” [45]. This geography takes the form of shapes, colors, weaves and installations that articulate emotions, physical suffering, myths and taboos inscribed in the flesh [46,47]. This dimension offers knowledge on the behaviors of the subjects and is based on the fact that subjects respond to a certain normative order. 

They conduct themselves or inscribe their acts in the framework that it indicates. There is a gap between what is ideal and what is procedural […] Without that, the subject and the necessary distance on which it is constituted would disappear [43].

The iconographic, as a motivated cultural product, is interesting; it is constructed to produce an effect, namely, a discourse with the intention of expressing the meanings of a creation that opens interpretative doors to us [48].

In the corporal map of the women sex worker, red is self-interpreted as negative emotions, such as displeasure and disgust when interacting with the Miner Man. Pleasure, in red, represents performance for the satisfaction of the Miner Man. In this sense, the word “pleasure” written in red symbolizes emotions that hide a pure and true inner self [49]. Along the same lines, the black that outlines the vulva and pelvis would be the graphic manifestation of the split between the intimate site of pleasure and its representation for the client: the black shows the duality between illuminated and dark/hidden spaces [50].

The possibilities of the graphic expressions (shape, weave, color, textures, etc.) within the different methodological models are ample. For example, the human figure drawing (HFD) technique is a projective method from the field of individual clinical psychology [51,52] used for diagnosing adults and children. Based on culturalist studies, the merit of research that recovers historical documents graphed as valid records from the past is recognized and conceived as ideological constructions and as forms of human communication. The anthropology of the graphic communications media covers two types of research, specifically studies of reception that explore the impact of the graphic media on a culture [53,54]; and the study of how people, generally not Western, make their own productions [55].

The projective dimension comprises a set of symbolic products and arises based on the amalgam of the narrative and graphic dimensions. In this dimension, special attention is maintained on the immediate plane from where conflicts are elaborated, which take shape on the corporal map as texts, dialogues, mandates, reproaches and obstacles that impede the advancement toward transformations and subjective wellbeing and include ruptures and liberations [42].

### 5.7. Interpretation of the Documental Corpus: The Intertextual Arrangement

**Figure 5:**
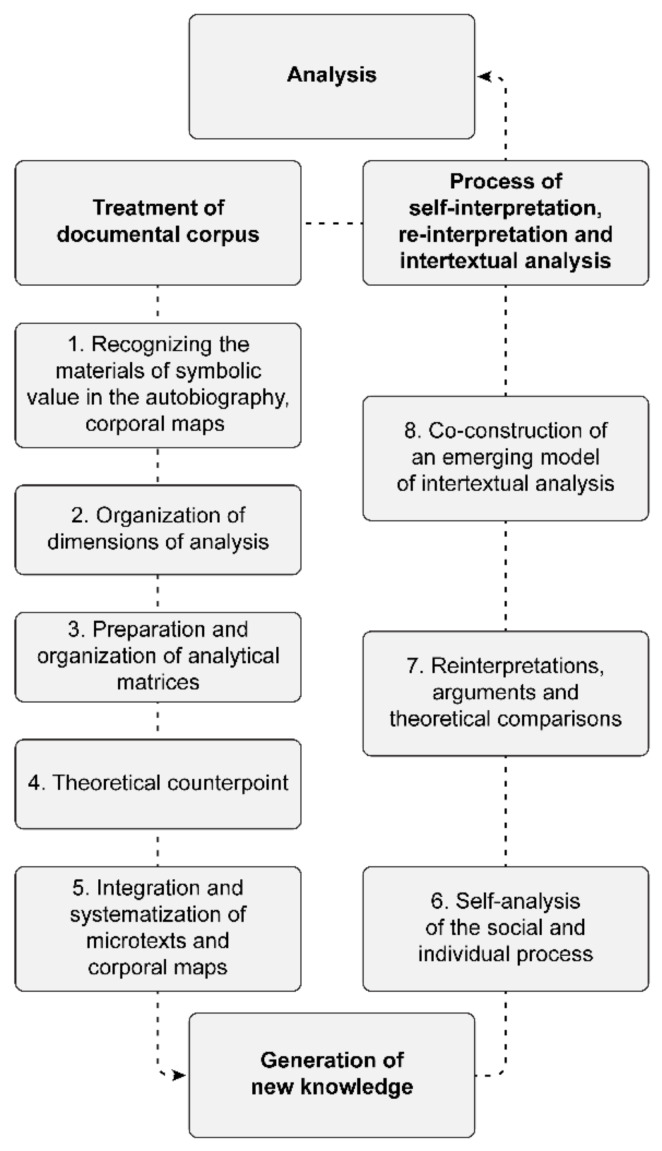
Example of interpretation of the documental corpus. Source: Self-created.

The “analysis” that accompanies the procedure (see Figure 5) is applied progressively in each stage of the production of symbolic materials. This way of proceeding approaches the posture of the anthropological field. This “analysis” is conducted with the participants throughout the research and consists of progressively constructing with them a representation of the culture incarnated in their bodies.

For the treatment of all the materials collected in the process, work is performed based on an intertextual understanding, which is inspired by the works of Kristeva [56], Derrida [57], Foucault [58] and Barthes [59]. These authors use intertextuality to provide an account of the multiple possibilities of language games. Barthes, for example, discusses an ideal of textuality where there is an abundance of networks that interact, without anyone being able to impose itself on the rest. The text that is produced using intertextuality is a galaxy of meanings and not a structure of meanings; the text has no beginning, but it does have various ways of access, without any of them being able to be qualified as the first. Furthermore, the codes that are mobilized extend as far as seen; they are indeterminable. In addition, the systems of meanings can be imposed on this absolutely plural text, but their number is never limited since it is based on the infinity of language [57].

The analysis is produced by entering from the microtexts the set of verbal accounts and written autobiographies and articulating the idea of establishing an intertext with the graphs of the corporal maps; their textures, color and icons are integrated into the emotions gathered in the process. It is proposed that the search not be hierarchical by playing with the findings such as in a network of meanings. In this game, the technique of interpretation incorporates some contributions from discourse analysis theory [43,60,61] and breaks with the structuralist element of its classic source. For the drawings, work is conducted with contributions from Bateson [62], and it incorporates some elements of the projective [47] and anthropological techniques [46,63]. (The work of Gregory Bateson [62] emphasizes the role of symbolic interactions and the value of cultural meanings in human interactions. Machover’s contribution consists of making a proposal based on the psycho-dynamic perspectives for analyzing projective elements of intrapsychic perspectives in the use of graphic methodologies. Finally, the contributions of Roland Barthes stand out for revealing the value of color, image, and light in artistic_cultural creations where the author grants high symbolic value to the graphic content. He also contributes with important methodological ruptures in the application of intertextuality for the analysis of cultural products).

Although we propose that this theoretical view provides contributions to the need for generating a work methodology that rescues the languages of the body, we suggest that the theoretical election at the time of performing the interpretive act remain at the free choice of the person who performs the process accompanied by the person who educates or applies the model. The idea is that in the entire interpretive process, the creativity that it allows is not lost, but above all, the intersubjective character is associated with the interpretation of the material.

With this model applied to education and social research, in its objective of producing new knowledge on corporeality, it is proposed to work on the material for interpretation and analysis in the following two moments: (1) treatment of the production: the order of the accounts and materials; and (2) interpretation of the discourses: the organization of topics and/or emerging categories on articulating grids or matrices. The first moment, treatment of the production, has the following five stages:

- Stage (1). Reordering of the materials of symbolic value: process of analysis and interpretation: overall understanding of the autobiographical account, selection of significant fragments or micro texts according to the search criteria or dimensions to be studied;
- Stage (2). Organization of the biographic events: organized on grids, the microtext of the account that accompanies the corporal map is integrated to attempt to establish a significant intertextual network;
- Stage (3). Preparation of grids: integration of elements from the drawing of the corporal map: a knot is set up comprised of color and the shape of the image and icons, with accounts on a grid or matrix that enable organizing the intertextual analysis;
- Stage (4). Interpretation of discourses: A theoretical counterpoint is located according to the disciplinary emphasis of utility for the production of knowledge that articulates the voices of the protagonists, the researcher and the theoretical base; and
- Stage (5). Systematization of Findings: during the entire process, the findings invite interpreting and making a nexus between one language and another, but when all the texts are interwoven, a level of interpretive complexity is reached. This level facilitates making a counterpoint with theoretical elements that are coherent with the searches, which grant greater consistency and density to the analysis, to respond to questions and objectives in the case of research.

The entire process leads to the preparation of the final conclusions or reflections. In this stage, the researcher’s work will consist of resolving the initial questions on the problem and discussing how the application of this model has allowed understanding and generated new knowledge.

## 6. How to Work with Educators on Sexuality

Intertextual workshops should be understood as part of a group process of reflection and participatory education and not as therapeutic strategies. In this sense, if cases are detected that require specialized attention from health professionals, the recommendation is to refer them to such attention.

As a facilitator, you are a coprotagonist in this process with the group. Your personal style and your skills in listening to the group, building a safe and respectful climate, and maintaining motivation will be key aspects throughout the sessions.

### 6.1. Workshop Sessions

The activities have been designed to be conducted as a comprehensive program.

Programs that have been evaluated in various countries have shown that the completion of more than 10 sessions has a greater impact on young people. Although it has been seen that isolated sessions also have some impact, the implementation of a multisession group process is recommended.

As mentioned in other sections of this article, it is recommended to work with groups of 6 to 14 people. It is also proposed to address the topics of each session by focusing on them in the most concrete way possible. In this sense, it is suggested to work on the topics in the here and now (present) of the experience (individual, group, family, institutional, community).

The suggestion to work with smaller or larger-size groups will depend on the capacity of the monitors to guide–accompany the process of listening steadily to each person participating in the group. For that reason, smaller-size groups are suggested, so there is cohesion, empathy and support for each story.

#### 6.1.1. Basic Conditions to Conduct the Workshop

A minimum of 55 min and a maximum of 100 min is suggested for each session. Different factors of each group or the context can cause these times to increase or decrease. Therefore, the activities can be adapted to the time that the facilitators actually have available and consider to be appropriate for conducting each session.

- Conduct activities in a spacious, ventilated and illuminated space, with as few distractions as possible, where participants can circulate and simultaneously talk in private.
- During the sessions, offer some food. Drinks and food are highly valued by youth and help to keep them engaged in the group process.

#### 6.1.2. Materials for the Workshop Sessions

- Booklets are required to take notes and write biographies, which are key parts of the process. (See Table 1. Planning Example)

#### 6.1.3. For the Beginning of the Session

At the beginning of the activities, it is useful for each participant to comment on “how they are when they arrive” at the session, whether something significant has happened during the week, or what they have been thinking, feeling or observing after having participated in the previous activity. Sharing their experiences is usually sufficient to provide a good emotional climate for the session.

#### 6.1.4. Notes for Facilitators

This activity works on the distinctions between sex (the biological differences between men and women) and gender (the sociocultural constructions of masculine and feminine). Unlike our sex, which has generally immutable characteristics, gender definitions change from generation to generation, from one culture to another and within different socioeconomic and ethnic groups, among other groups.

#### 6.1.5. For Planning the Workshops

Planning is proposed, which can be adjusted according to the characteristics of the groups. It is essential that the described process be experienced by the facilitators and subsequently applied to the students by adapting to the cultural context and their bodily practices. From the general objective to develop self-care, autonomy and sexual empowerment through the expression of corporality, we work with 6 to 14 people.

The table below is a guide for the monitors to organize and apply the methodological model in a workshop format, detailing the number of sessions, the activities to be carried out, the estimated time, and the materials necessary for completing the process. It should be pointed out that this is a guide that can be adapted according to the context territory. Additionally, the number of sessions can be modified depending on the time the facilitator has available.

## 7. Conclusions

The works based on the biographical method have traditionally left voids of knowledge on the discourses of the sexual and emotional experiences that the body produces. For the purpose of providing a biographical method, we have systematized this proposal, which facilitates understanding the process of constructing a subject on the materiality of the body. With these tools it is possible to promote bodily self-awareness and prevention in matters of sexuality, by encouraging self-care. Taking into account that in building body schematics, elaborating a history of the body itself and the emotions that come with it, subjective images are articulated with identity and psychosexual processes accumulated throughout life, which position the body in a protagonist role in the personal biography.

In life stories and accounts, although representations are constructed on the experiences of the body, the subject speaks and prepares the discourse on it by remaining silent on corporality and leaving it in its own language. Therefore, the methodological model of corporal maps for sex education proposes that recovering the languages that are given to a body in their sexual, psycho-emotional, cultural, and social and gender domains open passages to the complex relationships among self-image, normative cultural prescriptions and social contexts.

In this way, education on sexuality under this methodological model facilitates deconstructing gender positions adopted based on the symbolic order expressed in the body and can also offer information that facilitates the elaboration of new interpretations from this order that enable us to generate processes of reflection on the students’ biographies and simultaneously offer options for reconstructing subjectivity.

All of these articulations require researchers to go beyond what has been reached by the projective tools in clinical diagnosis in psychology, where the subject is evaluated in an asymmetric relationship. These articulations also enable a progression in biographical research with a technique that contributes relevant information on corporal experiences in their semiotic–material complexity.

With Corporal Maps, the protagonist of the biography becomes the author of the interpretation. Bodies are therefore not treated as “objects” of specific studies for their classification as healthy or ill (which in a certain way, would lead to reinstalling Cartesian dualism) but rather are recognized in broad constitutive and unavoidable dimensions of any social practice. The foregoing makes sense in the Latin American sociopolitical and cultural contexts, where socioemotional experiences are constructed in highly globalized and fragmentary contacts of the subject’s experience.

In societies such as in Latin America, with gender relationships organized mainly on hierarchies of power, we construct ourselves on the one hand from metaphors, fictions, remnants and sutures, and on the other hand, we are interpolated to consume and produce. These demands trap the subject in a paradoxical existence that many times leaves the subject silent up to the point of pain or destruction. In this sense, this methodological model offers strategies for understanding how the culture is incarnated based on sociocultural discourse and is interweaved in the intrapsychic world; this cultural understanding is involved in interpersonal relationships, depending on the type of prominence that each subject acquires, and is anchored to a set of current societal norms. This model also opens an option for renewing the transdisciplinary dialogues among anthropology, sociology, psychology and psychoanalysis. This means that in listening to the languages of the body, we find a rich way of interpreting conflicts among the ideal normative guidelines of the culture and the experience of the intersubjective work with norms inscribed in the flesh.

However, similar to any model, it has limitations. With respect to the organization of the groups, it is not recommended for working with a high number of people and is optimal only with groups of 15 to 20 people per workshop. Among the procedures and their risks, it is important to avoid exploring aspects that transgress their intimacy and that expose the participants to the prejudices and judgements of the other group members. In this regard, for example, it is necessary to previously evaluate whether or not it is appropriate to have mixed spaces (men and women, children and adults).

Regarding educators, monitors or guides, they need to have training in the technique and its theoretical grounds for their development. In the process, they can contribute to identifying and managing the personal, family, community and institutional resources to which people can turn. It is important to attentively note people’s demands, needs and requirements for support and to transmit them at the pertinent times. In particular, it is necessary to attend to the demands for psychological support, and there need to be spaces that enable providing specialized attention.

An important consideration refers to the context in which the model is applied, since in the experiences in multicultural Andean environments, particularities were observed due to the different symbolic interpretations and assessments of the body and of social relations. For example, in the case of Andean world views, the body will possess meanings different from Westernized views [62], such as the case of the four phases of the “Andean life cycle” [63] (According to Bascopé [63] and Van Kessel [64], in the Andean lifecycle, the body reflects the worldview in the four dimensions of up, down, left and right. The birth space is located in the “left” part, which symbolizes the relationship with the origins of life and refers to the start of all living things. Growth is located in the “down” part in relation to the dimension of the conservation, restoration and recreation of everything created: the Pachamama represents masculine and feminine and the sense of fertility that existence gives. Death is located in the “right” part and is articulated with the sense of conclusion, completion, arrival, culmination, and a space for projection after concluding a stage of life. These representations inscribed or incarnated based on the linguistic community possess a fundamental weight in the corporal socialization of the subjects. Although it has some processes in common with the Eurocentric Western culture, Andean corporal worship and representations generate dual interpretations linked to the prevailing deities in its imagery that are not found in Western culture [65]).

These cultural particularities must finally be a central element in the organization of the strategies for the different applications. A recommendation that contributes to the development of the model will finally consist of working to emphasize the orality and the graphic phase introduced with other methodologies appropriate for the group’s sociocultural contexts.

## Figures and Tables

**Table 1:** Workshop planning example.

Session	Description	Minutes	Materials
Specific objective 1: Recover significant events from the biographical path	Framing: describe the objective and timing of the process, presentation and commitment Exposition of the corporal map already done to exemplify Elaboration of the lines of gender events in three stages: early childhood (0–6 years); childhood (6–12); and adolescence (13–present) Voluntary expression of significant events of gender construction (happy and sad) in group conversation Close with comments on the experience. Commitment 2nd session. Write about what “I believe about me, who I am, what I do, how I see myself, my personality”	20 min 20 min 15 min	Informed consent Cardboards, pencils and markers
	Total time: 55 min		
Session 2 Specific objective 2: Identify the socioemotional processes linked to the construction of the body self-image	Summary of the previous session and questions about the state of mind Beginning of an extended autobiography determined by each adolescent I will write about who I am, what I do, how I see myself and how I describe my gender construction Then, I will write about how I think others see me, what they think of my personality and what I do for self-care in sexuality and in gender interactions Closing. Recovery of the most positive aspects	10 min 30 min 15 min	Paper and pencils
	Total time: 55 min		
Session 3 Specific objective 3: Identify the socioaffective processes linked to the construction of the corporal self-image	Summary of the previous session. Questions about the mood state Continue with the completion of the autobiography. The significant events of body experience and body self-care Conversation about the emotions associated with the corporal experiences and gender experiences: positive and negative. What I think others think about my body, what I feel and think about self-care and my body Preparation of the body diagrams of the participants on paper Close with comments on the experience and commitment for the next session: painting of the bodies on paper	15 min 30 min 20 min 10 min	Paper and pencils Tracing or craft paper for each participant Body maps to exemplify methodology
	Total time: 75 min		
Session 4: Specific objective 4: Identification of the experiences of corporal self-care through the construction of an intertextual map	Summary of the previous session and questions about the state of mind Instructions for work on body maps. Painting and free writing on paper Prepare in pairs a scheme of their own natural body size. Lying on the paper or cloth, the partner draws the outline of the body, and then, they trade places Colors, signs, objects are freely chosen to illustrate the subjective experience with the body on a map to show the geography of the bodies Close with comments on the experience 15 min	15 min 30 min 45 min 15 min	Papers or fabrics of individual body size, pencils, markers, temperas, brushes, objects to insert, free choice
	Total time: 110 min		
Session 5 Specific objective 5: Identification of the body experiences, stories, colors, symbols that represent corporality on the map	Summary of the previous session and questions about the state of mind Voluntary exhibition of finished maps, individual comments on its creation, self-care, and the emotional sexual body Group comments about the experience Final closing free	10 min 45 min 30 min	Craft paper or individual tracing paper, pencils, markers, temperas, brushes, luster paper
	Total time: 80 min		

## Data Availability

The data supporting the findings of this study are available on request from the corresponding author (J.S.S., FONDECYT 1180079). The data are not publicly available due to their containing information that could compromise the privacy of research participants.
